# JAK2 inhibitor TG101348 overcomes erlotinib-resistance in non-small cell lung carcinoma cells with mutated EGF receptor

**DOI:** 10.18632/oncotarget.3685

**Published:** 2015-03-29

**Authors:** Fu-quan Zhang, Wen-tao Yang, Shan-zhou Duan, Ying-chen Xia, Rong-ying Zhu, Yong-bing Chen

**Affiliations:** ^1^ Department of Cardiothoracic Surgery, the Second Affiliated Hospital of Soochow University, Suzhou, Jiangsu, China

**Keywords:** non-small cell lung cancer, TG101348, erlotinib, drug resistance, cancer therapy

## Abstract

Non-small cell lung cancer (NSCLC) patients with epidermal growth factor receptor (EGFR) mutations are responsive to EGFR-tyrosine kinase inhibitor (EGFR-TKI). However, NSCLC patients with secondary somatic EGFR mutations are resistant to EGFR-TKI treatment. In this study, we investigated the effect of TG101348 (a JAK2 inhibitor) on the tumor growth of erlotinib-resistant NSCLC cells. Cell proliferation, apoptosis, gene expression and tumor growth were evaluated by diphenyltetrazolium bromide (MTT) assay, flow cytometry, terminal deoxynucleotidyl transferase biotin-dUTP nick end labeling (TUNEL) staining, Western Blot and a xenograft mouse model, respectively. Results showed that erlotinib had a stronger impact on the induction of apoptosis in erlotinib-sensitive PC-9 cells but had a weaker effect on erlotinib-resistant H1975 and H1650 cells than TG101348. TG101348 significantly enhanced the cytotoxicity of erlotinib to erlotinib-resistant NSCLC cells, stimulated erlotinib-induced apoptosis and downregulated the expressions of EGFR, p-EGFR, p-STAT3, Bcl-xL and survivin in erlotinib-resistant NSCLC cells. Moreover, the combined treatment of TG101348 and erlotinib induced apoptosis, inhibited the activation of p-EGFR and p-STAT3, and inhibited tumor growth of erlotinib-resistant NSCLC cells *in vivo*. Our results indicate that TG101348 is a potential adjuvant for NSCLC patients during erlotinib treatment.

## INTRODUCTION

Lung cancer is the major cause of cancer death worldwide [1, 2]. More than 80% of lung cancer patients are non-small cell lung cancer (NSCLC) [1, 2]. NSCLC is characterized by a number of gene point mutations that disrupt the normal growth and survival of the lung epithelium. Overexpression of epidermal growth factor receptor (EGFR) has been shown in about 19%~51.4% of NSCLC patients [3, 4] and has been related with poor prognosis in NSCLC patients [5, 6]. EGFR, a receptor tyrosine kinase, is aberrantly activated in several solid tumors, especially in NSCLC [2]. Studies have shown that EGFR-activated cell signaling plays important roles in lung tumorigenesis and tumor progression [7, 8]. Therefore, targeting inhibition of EGFR signaling has been demonstrated to be an effective approach for NSCLC treatment [9-11].

Studies have shown that approximately 70% of NSCLC patients present with somatic mutations in the exons of the EGFR gene. These EGFR mutations include the deletion mutation of DE746-A750 in exon 19 and the leucine-to-arginine substitution at position 858 (L858R) in exon 21 of the *EGFR* gene [12]. The NSCLC patients with these EGFR mutations respond well to the treatment with small-molecule EGFR tyrosine kinase inhibitors (EGFR-TKIs), including erlotinib [13, 14]. However, most patients, even those markedly responsive to initial treatment, develop resistance to EGFR-TKIs later [15]. Recent studies have shown that several mechanisms are involved in the development of resistance to EGFR-TKIs: secondary mutations of EGFR (e.g. T790M in exon 20 and D761Y, in exon 19) [12], amplification of MET [16], persistent survivin overexpression [17, 18], constitutive activation of JAK2/STAT3 [19-22] and the activation of Ras phosphatidylinositol-3 kinase (PI3K)/Akt pathways [23, 24]. Developing new agents to overcome the EGFR-TKI resistance would be important for long-term treatment in NSCLC patients.

EGFR signaling, involved in multiple intracellular pathways, promote cell proliferation and suppress apoptosis [23, 25]. Constitutive activation of STAT3 is a common characteristic in many solid tumors including NSCLC. Although STAT3 activation is frequently attained by JAK2 somatic mutations in hematologic malignancies, similar mutations are not commonly seen in solid tumors. Previous studies have shown that STAT3 activation in solid tumors is commonly induced by hyperactive growth factor receptors or autocrine cytokine signaling. Constitutive STAT3 activation has been proposed to play an important role in resistance to various small-molecule therapies that target oncogene signaling pathways. Recent studies have demonstrated that STAT3 is constitutively activated in human NSCLC samples and in a variety of NSCLC lines, independent of activating KRAS or tyrosine kinase mutations [21]. NSCLC cells secrete IL-6 and consequently activate STAT3 via autocrine mechanism [26]. The EGFR-TKI resistant NSCLC cells express constitute activation STAT3 signaling [20]. These data indicate that constitute activation of JAK2/STAT3 signaling plays critical roles in mediating the resistance to EGFR-TKIs. Genetic or pharmacologic inhibition of the gp130/JAK2 signaling pathway disrupts activation of STAT3 [21]. Treatment of NSCLC cells with the JAK1/2 inhibitor suppresses growth in soft agar and xenograft assays [21]. Therefore, targeting inhibition of JAK2/STAT3 may be a new treatment approach in NSCLC patients with EGFR-TKIs resistance.

TG101348 is a small-molecular highly selective ATP-competitive JAK-2 inhibitor [27, 28]. TG101348 inhibits the proliferation of human erythroblast leukemia (HEL) cell line that harbors the JAK2V617F mutation as well as a murine pro-B cell line expressing human JAK2V617F [27, 28]. Recent studies have shown that TG101348 specifically decreases Hodgkin lymphoma and mediastinal large B-cell lymphoma growth *in vitro* and *in vivo* [29]. Clinical trials have shown that TG101348 is well tolerated and produces significant reduction in disease burden and durable clinical benefit in patients with myelofibrosis [30]. However, the potential effect of TG101348 combined with erlotinib for NSCLC treatment is unknown.

In this study, the effect of TG101348 on EGFR-KI-resistant NSCLC cells *in vitro* and *in vivo* was determined. TG101348 was found to significantly increase the cytotoxicity of erlotinib, enhance erlotinib-induced apoptosis, and inhibit the tumor growth in EGFR-TTKI-resistant NSCLC cells. Our results suggest that TG101348 is a promising treatment agent for NSCLC patients resistant to erlotinib.

## RESULTS

### TG101348 induces apoptosis of NSCLC cells

**Figure 1:**
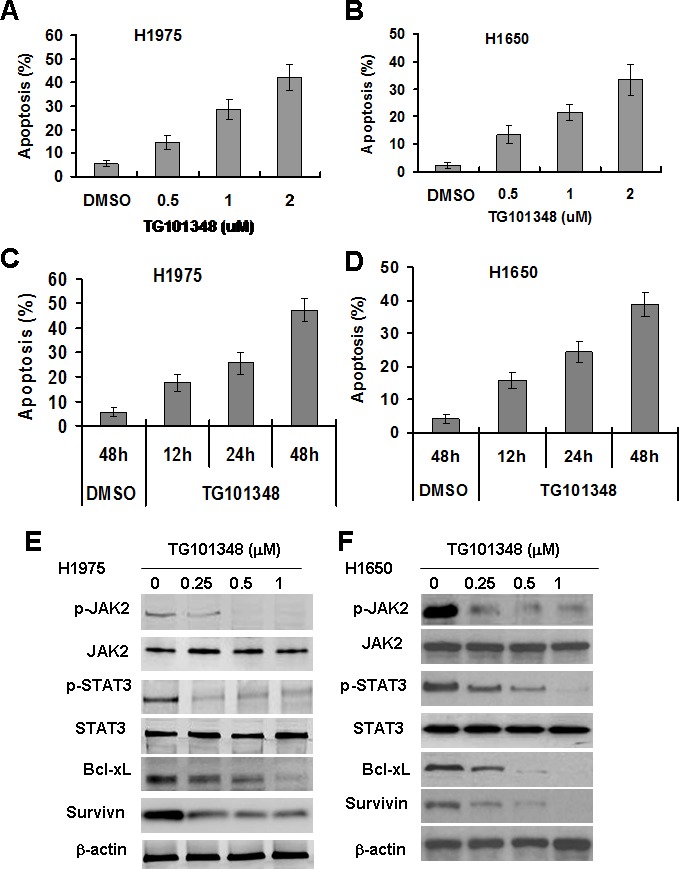
TG101348 induces apoptosis and inhibits JAK2/STAT3 signaling in NSCLC cells with EGFR-mutation (**A**-**D**) TG101348 induces apoptosis of NSCLC cells. H1975 cells (A, C) and H1650 cells (B, D) were treated with erlotinib or TG101348 at the indicated doses (A, B) and time (C, D) for 48 hours. Cell apoptosis was determined by TUNEL. The data are means ± SD of three independent experiments. (**E**-**F**) TG101348 inhibits JAK2/STAT3 signaling in NSCLC cells. H1975 cells (E) and H1650 cells (F) were treated with TG101348 at the indicated doses for 24 hours. Protein expression was determined by Western Blot.

Previous studies have shown that the aberrant activation of JAK2/STAT3 signaling was found in NSCLC tumors [21]. It has been reported that PC-9 cells is erlotinib-sensitive and H1650 cells and H1975 cells are erlotinib-resistant [31]. We found that the levels of IL-6, p-JAK2 and p-STAT3 in H1975 and H165 cells were higher than in PC-9 cell (Supplementary Fig. 1A and 1B). Further, knockdown of STAT3 sensitized H1975 cells to erlotinib-induced apoptosis (Supplementary Fig. 2A and 2B), confirming that the IL-6/JAK2/STAT3 pathway is involved in mediating resistance of erlotinib. To determine the effect of TG101348 on apoptosis of NSCLC cells, PC9, H1975 and H1650 were used. TG101348 treatment markedly induced apoptosis in all three NSCLC cell lines dose-dependently (Fig. 1A-1D) and time-dependently (data not shown). This study focused on whether TG101348 inhibited JAK/STAT3 signaling in these NSCLC cells, and also studied the expression of molecules of JAK2/STAT3 signaling in NSCLC cells treated with TG101348. TG101348 treatment decreased the level of p-JAK2 and p-STAT3 in both H1975 cells and H1650 cells in a dose-dependent manner (Fig. 1E-1F). TG101348 treatment inhibited expression of apoptosis-related protein Bcl-XL, Bcl-2, survivin, XIAP, and resulted in the cleavage of caspase 3 (Fig. 1E-1F, Supplementary Fig. 3). The results indicate that TG101348 induces apoptosis in EGFR-mutant NSCLC cells through the inhibition of JAK2/STAT3 signaling.

### TG101348 sensitizes erlotinib-resistant NSCLC cells to the cytotoxicity of erlotinib

**Figure 2:**
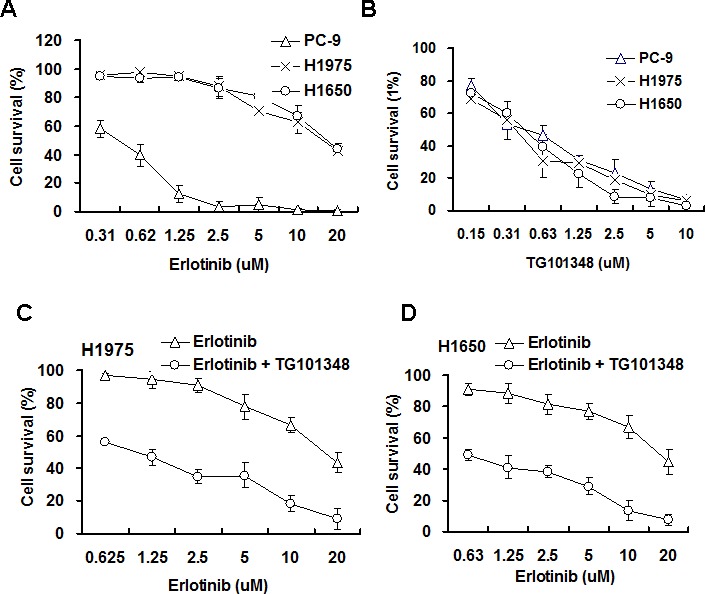
TG101348 sensitizes NSCLC cells to the cytotoxicity of erlotinib (**A**-**B**) Erlotinib and TG101348 inhibit cell proliferation. PC-9, H1975 and H1650 cells were treated with erlotinib (A) and TG101348 (B) at the indicated doses for 72 hours. (**C**-**D**) TG101348 sensitizes the cytotoxicity of erlotinib to NSCLC cells. H1975 cells (C) and H1650 cells (D) were treated with erlotinib at the indicated doses alone or combined with TG101348 (1 μM) for 48 hours. Cell proliferation was determined by MTT. The data are means ± SD of three independent experiments. (*p < 0.05, compared to erlotinib treatment alone).

EGFR-mutant NSCLC H1975 and H1650 have been reported to be erlotinib-resistant [19, 21, 32]. The effect of TG1010348 on cell proliferation in EGFR-mutant NSCLC was first studied to determine the effect of TG1010348 on the resistance to erlotinib in these cells. Results showed that erlotinib dramatically inhibited cell proliferation of PC-9 cells. The IC50 of erlotinib was approximately 0.58 μM for PC-9 cells (Fig. 2A). H1975 cells and H1650 cells were more resistant to erlotinib than PC-9 cells. The IC50 of erlotinib was 14.3 μM and 17.6 μM for H1950 cells and H1650, respectively (Fig. 2A). The results further confirmed that PC-9 cells is erlotinib-sensitive and H1650 cells and H1975 cells are erlotinib-resistant, consistent with previous studies [31]. No significant difference in inhibition rates were found among the three NSCLC cell lines when treated with TG101348 (Fig. 2B), indicating that TG101348 possesses strong inhibition in erlotinib-resistant NSCLC cells than in erlotinib-sensitive NSCLC cells. H1975 cells and H1650 cells were treated with a combination of erlotinib and TG101348 to study whether TG101348 reverses erlotinib resistance. When combined with TG101348 (1.0 μM), erlotinib significantly increased the inhibition of cell proliferation in H1975 cells (Fig. 2C) and H1650 cells (Fig. 2D), compared with erlotinib treatment alone. These results suggest that TG101348 has additive effect on erlotinib cytotoxicity to NSCLC cells.

### TG101348 enhances erlotinib-inhibited colony formation of EGFR-mutant NSCLC cells

**Figure 3:**
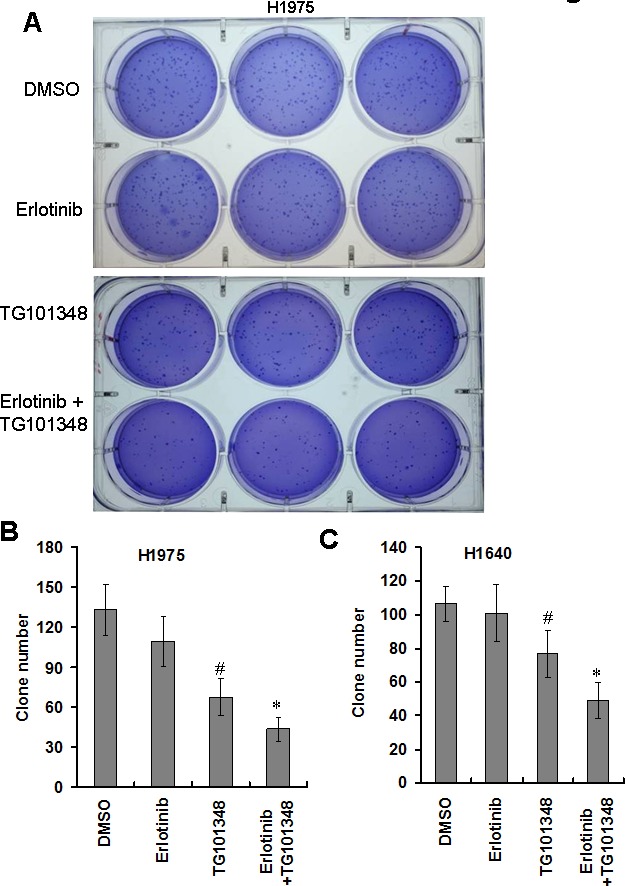
TG101348 inhibits colony formation of NSCLC cells (**A**) Colony forming ability of H1975 in soft agar. H1975 cells were plated in 0.3% agar with 0.25 μM TG101348 and cultured for 2 weeks. Representative photos were taken at 2 weeks after the cell seeding. (B-C) Quantification analysis of clone number. H1975 cells (**B**) and H1650 cells (**C**) were plated in 0.3% agar for 2 weeks. Clone numbers were counted under microscope. Data represents the pooled means ± SD of 3 independent experiments, each consisting of triplicate samples for a total n = 9 for each treatment. *p < 0.01 compared to single treatment. #*p* < 0.01 compared to DMSO control.

We further investigated the effect of TG101348 on cell transformation. Soft agar assay is a classic approach to determine cell transformation *in vitro*. The result showed that H1975 cells could form a large number of colonies in soft agar, and erlotinib treatment slightly inhibited colony formation (Fig. 3A-3B), suggesting that H1975 cells are resistant to the inhibition of anchorage-independent growth of erlotinib. TG101348 treatment significantly reduced colony formation of H1975 cells (Fig. 3A). Quantification showed that the treatment with TG101438 reduced the number of colonies by 51 ± 3.6 %, compared to DMSO-treated colonies (Fig. 3B). This result suggests that TG101438 alone inhibits anchorage-independent growth of H1975 cells. The combination treatment of erlotinib and TG101438 exhibited the strongest inhibition of colony formation of H1975 cells than either monotherapy of erlotinib or TG101438 (Fig. 3B). Although erlotinib monotherapy did not significantly reduce the number of colonies in H1640 cells (Fig. 3C). TG10138 as monotherapy significantly reduced the number of colonies. However, the combination of erlotinib and TG101348 reduced the number of colonies in H1640 cells (Fig. 3C). These results indicate that TG101348 enhances erlotinib-inhibited anchorage-independent growth and colony formation of EGFR-mutant NSCLC cells.

### TG101348 enhances erlotinib-induced apoptosis of EGFR-mutant NSCLC cells

**Figure 4:**
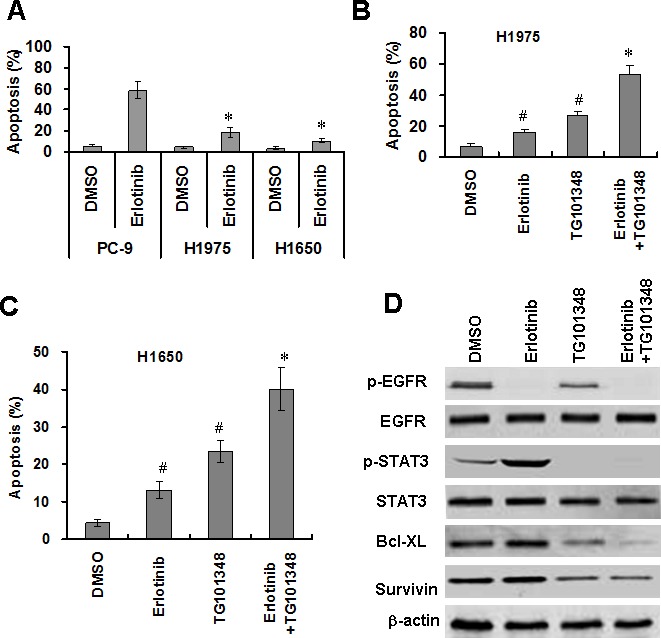
TG101348 enhances erlotinib-induced apoptosis in NSCLC cells (**A**) erlotinib induced apoptosis in NSCLC cells. PC-9, H1975 and H1650 cells were treated with erlotinib (2.5 μM) for 72 hours. Apoptosis was determined by TUNEL. *p < 0.01, compared to PC-9 cells. (**B**-**C**) TG101348 enhances erlotinib-induced apoptosis in erlotinib-resistant NSCLC cells. H1975 cells (B) and H1650 cells (**D**) were treated with TG101348 (1 μM), erlotinib (2.5 μM), or a combination of TG101348 (1 μM) and erlotinib (2.5 μM) for 48 hours. Apoptosis was determined by TUNEL. The data presented are means ± SD of three independent experiments. ^#^p < 0.01, compared to DMSO treatment; *p < 0.01, compared to TG101348 or erlotinib treatment alone. (D) TG101348 regulates expressions of EGFR and apoptosis-related proteins. H1975 cells were treated with TG101348 (1 μM), erlotinib (2.5 μM) or their combination for 48 hours. Protein expressions were determined by Western Blot.

To assess the effect of erlotinib on apoptosis in EGFR-mutant NSCLC cells, NSCLC cells were treated with erlotinib (1 μM) for 48 hours. Erlotinib treatment markedly induced apoptosis of PC-9 cells, but only slightly induced apoptosis of H1975 cells and H1650 cells (Fig. 4A). These results suggest that H1650 cells and H1975 cells are resistant to erlotinib-induced apoptosis. To investigate whether TG101348 increased erlotinib-induced apoptosis of erlotinib-resistant cells, H1650 and H1975 cells were treated with TG101348, erlotinib or their combination. The combination treatment significantly increased apoptosis in both H1975 cells (Fig. 4B) and H1650 cells (Fig. 4C) compared to either TG10138 or erlotinib as monotherapy. The results indicate that TG101348 enhances erlotinib-induced apoptosis of EGFR-mutant NSCLC cells.

To determine the underlying mechanisms by which TG101348 enhances erlotinib-induced apoptosis in EGFR-mutant NSCLC cells, we determined the effect of TG101348 on EGFR and STAT3 signaling. Western Blot showed that erlotinib decreased p-EGFR level but slightly increased p-STAT3 expression (Fig. 4D). TG101348 downregulated pSTAT3 in H1650 cells (Fig. 4D). The combination treatment of TG101348 and erlotinib inhibits the activation of both p-EGFR and p-STAT3 (Fig. 4D).

Results showed that erlotinib did not affect expression of survivin and Bcl-XL in H1650 cells (Fig. 4D). In contrast, TG101348 markedly decreased expression of survivin and Bcl-XL. Moreover, the combination treatment of TG101348 and erlotinib exhibited the strongest inhibition of (Fig. 4D). These results suggest that the inhibition of expression of survivin and Bcl-XL contributes to the TG101348-induced decrease in erlotinib-resistance in EGFR-mutant NSCLC cells.

### TG101348 potentiates the anti-tumor effect of erlotinib in NSCLC *in vivo*

**Figure 5:**
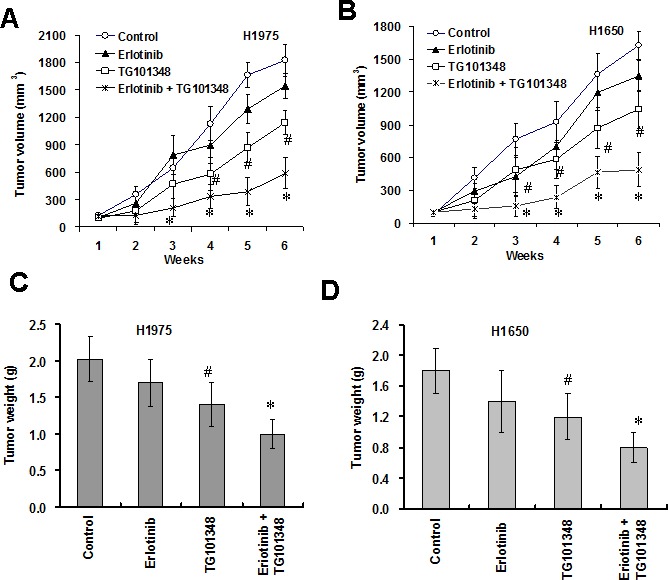
TG101348 potentiates the anti-tumor effect of erlotinib *in vivo* (**A**-**B**) TG101348 enhances the inhibition of tumor growth of erlotinib *in vivo.* H1975 cells (A) and H1650 cells (B) were injected s.c. into the flanks of athymic nude mice. The tumor-bearing mice received TG101348, erlotinib or their combination treatment for 5 days. Tumor volume was measured weakly for 6 week after the treatment. The tumor volume presented is means ± SD of five mice. (**C**-**D**) Tumor weight was weighed at the last day of the experiment above. Tumor weights presented are means ± SD of five mice. *p < 0.01, compared to TG101348 or erlotinib treatment alone; #p < 0.01, compared to DMSO treatment.

To investigate whether TG101348 enhances the ant-tumor action of erlotinib *in vivo*, we determined the effects of TG101348 and erlotinib on tumor growth in a xenograft mouse model of H1650 cells and H1975 cells. The results showed that erlotinib slightly inhibited tumor growth while TG101348 significantly inhibited tumor growth (Fig. 5A-5B, supplementary Fig. 4) and reduced tumor weight (Fig. 5C-5D) in H1975 cells and H17650 cells compared to the control treatment. The combination treatment of TG101348 and erlotinib exhibited stronger inhibition of tumor growth and reduction of tumor weight than TG101348 or erlotinib monotherapy (Fig. 5A-5D). These results suggest that TG101348 enhances the response of EGFR-mutant NSCLC to erlotinib and potentiates the antitumor effect of erlotinib *in vivo.*

### TG101348 enhance erlotinib-induced apoptosis and inhibit expression of STAT3 expression *in vivo*

**Figure 6:**
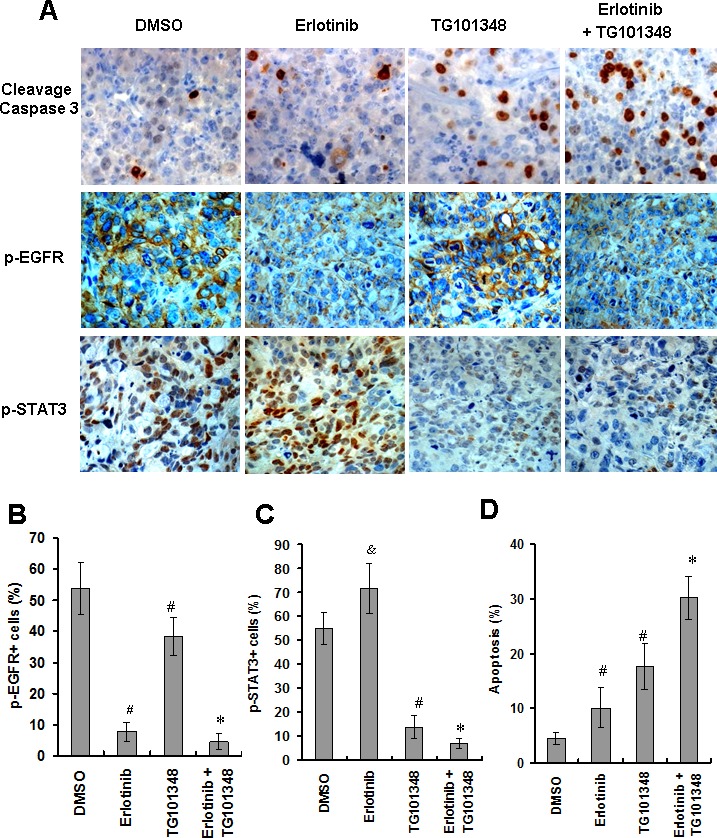
TG101348 enhances erlotinib-induced apoptosis and inhibition of gene expression *in vivo* (**A**) Tumor sections from the mice indicated in Figure 5 were subjected to cleavage caspase 3 staining (as an indicator of apoptosis), p-EGFR and p-STAT3 staining. Representative images are shown (Original magnification × 400). (**B**-**D**) Quantification of apoptotic index and gene expression was described in “Materials and Methods.” Data presented are means ± SD of five mice. ^#^*p* < 0.01, compared to DMSO group; ^*^p < 0.01, compared to single treatment DMSO.

We further determined the mechanisms by which TG101348 enhanced erlotinib-inhibition of tumor growth *in vivo*. Tumors collected from erlotinib-treated mice expressed lower levels of p-EGFR but higher levels of p-STAT3 than tumors collected from DMSO-treated mice (Fig. 6A-6C), suggesting that erlotinib treatment resulted in activation of STAT3 signaling *in vivo*. Tumors from the mice treated with TG101348 expressed significantly lower levels of p-EGFR and p-STAT3 compared to the tumors from the mice treated with DMSO. Moreover, tumors from the mice treated with combination therapy of TG101348 and erlotinib showed significantly lower levels of p-EGFR and p-STAT3 than those of mice treated with either monotherapy of TG101348 or erlotinib (Fig. 6A-6C).

Consistent with inhibition of p-EGFR and p-STAT3, cleavage caspase 3-positive cells (apoptotic cells) were significantly higher in the tumors from the mice treated with combination therapy of TG101348 and erlotinib compared to tumors from the mice treated TG101348 or erlotinib alone (Fig. 6A and 6D). These results indicate that TG101348 enhance erlotinib-induced apoptosis in NSCLC cells *in vivo*.

### The combination of TG101348 and erlotinib prolongs survival time of tumor-bearing mice

**Figure 7:**
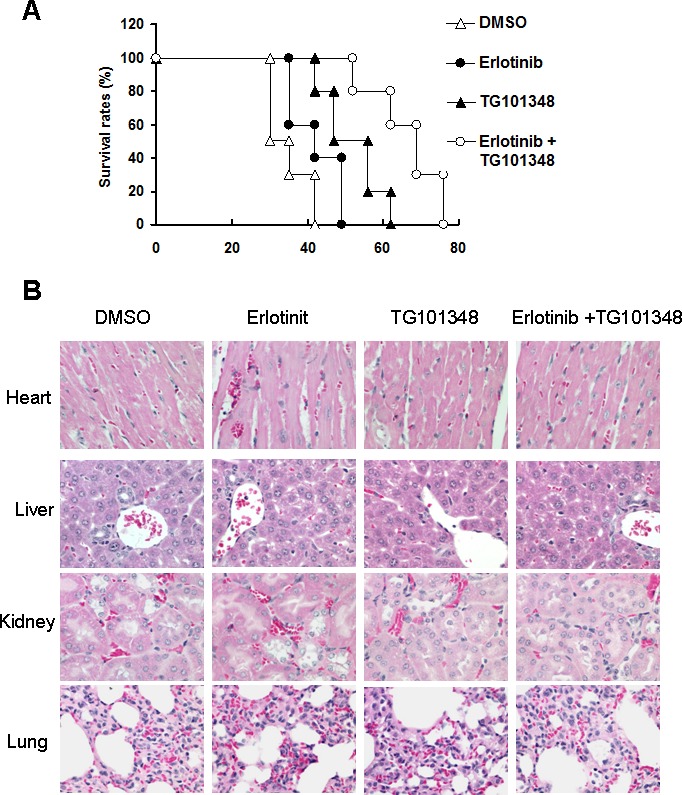
The combination of TG101348 and erlotinib prolonged the survival of mice (**A**) Tumor-bearing mice survival culture. H1975 cells were injected s.c. into the flanks of athymic nude mice. When tumor size reached 100 mm^3^, tumor-bearing athymic nude mice were treated with TG101348, erlotinib or their combination for 5 days. Mice were observed until the time point of death of all mice treated with the combination of TG101348 and erlotinib. (**B**) Histology analysis of main organs (heart, liver, kidney and lung) of mice treated indicated reagents 5 weeks after treatment (Original magnification × 200) are shown.

The effect of combined treatment of TG101348 and erlotinib on the long-term survival of mice bearing tumor was determined. The experimental end point was defined as the time when an entire group of mice died. Death was defined as natural death if tumor burden or tumor size was greater than 2.5 cm^3^. Mice challenged with H1975 cells died from tumor burden or were killed when they reached experimental end points. While mice treated with erlotinib monotherapy survived significantly longer than DMSO-treated mice (*P <* 0.05), all mice eventually died (Fig. 7A). 3 out of 10 mice treated with the combination therapy of G101348 and erlotinib were still alive without clinical symptoms 76 days after treatment. Furthermore, all mice survived significantly longer than mice treated with either TG101348 or erlotinib as monotherapy (*P < 0*.01) (Fig. 7A). Our results suggest that the combined treatment can prolong the survival time of tumor-bearing mice.

We evaluated the safety of combination treatment of TG101348 and erlotinib *in vivo* by detecting the pathologic alterations of four important organs from the mice 5 weeks after the treatment. Histology analysis showed that the tissues of heart, liver, lung and kidney in all mice did not show obvious pathologic alterations 5 weeks after treatment (Fig. 7B). These results demonstrate the safety of the combination treatment of TG101348 and erlotinib.

## DISCUSSION

In the study, we showed that TG101348 significantly enhanced erlotinib-inhibition of cell proliferation in EGFR-mutant NSCLC cells. The combination treatment of TG101348 and erlotinib obviously decreased the expressions of EGFR, p-EGFR, Bcl-xL and survivin, suppressed the activation of STAT3, induced apoptosis and inhibited tumor growth of EGFR-mutant NSCLC cells *in vivo*. Our results demonstrate that TG101348 could overcome erlotinib-resistance and enhance the anti-tumor action of erlotinib in EGFR-mutant NSCLC cells.

EGFR-targeted treatment has been shown to provide advantages over traditional chemotherapy for the advanced NSCLC [9, 14]. Studies have demonstrated that erlotinib is an effective drug for NSCLC patients with EGFR mutations [33-35]. However, most NSCLC patients finally develop resistance to EGFR-TKIs, including erlotinib. It is still merge to develop effective therapies for erlotinib-resistant patients. Studies have shown that the activation of JAK2/STAT3 signaling contributes to the development of erlotinib resistance [19, 26]. STAT3-mediated Akt activation resulted EGFR-TKI resistance in lung cancer cells [20]. Niclosamide reverses erlotinib resistance by supressing STAT3 activation in NSCLC [36]. Our results also showed that the the IL-6/JAK2/STAT3 pathway was activated in erlotinib-resistant H1975 and H1650 cells than in erlotinib-sensitive PC-9 cells. Knockdown of STAT3 increased erlotinib-induced apoptosis in H1975 cells. These data indicate that inhibition of JAK2/ STAT3 activation may overcome erlotinib resistance [37].

TG101348 has been shown to inhibit cell proliferation of primary hematopoietic cells and erythroblast leukemia (HEL) cell line that harbors the JAK2V617F mutation [27, 28]. TG101348 upregulates FOXO1 mRNA and protein expression in mediastinal B cell lymphoma cell lines MedB-1 and U2940 and inhibit the growth of mediastinal B cell lymphoma [38]. In addition, TG-101348 has been shown to modulate immune function [39]. TG-101348 preserves Treg numbers and thereby enhances the ratio of CD4(+) Tregs to CD8(+)CD25(+) effector T cells, and also reduces the production of IL-6 and TNF-α [39]. Data suggest that TG-101348 has multiple functions in tumor inhibition.

EGFR signaling has been recognized as an important regulator of cell proliferation and apoptosis [23]. A previous study has shown that a novel EGFR inhibitor promotes apoptosis of H1975 cells, but not that of H1650 cells [31]. In this study, we found that TG101348 potentiates erlotinib-induced apoptosis in both H1975 cells and H1650 cells. Recent data shows that the combination therapy of TG-101348 and BI-2536 (PLK1 inhibitors) synergistically targets oncogenic pathways [38] and that the combination therapy of TG101348 and imatinib (ABL kinase inhibitor) inhibits cell proliferation of BCR-ABL-positive chronic myeloid leukemia cells [40]. We also found that TG101348 could sensitize EGFR-mutant NSCLC cells to the cytotoxicity of erlotinib and enhance erlotinib-induced apoptosis of erlotinib-resistant NSCLC cells. Furthermore, the combination treatment of TG101348 and erlotinib significantly inhibited tumor growth of erlotinib-resistant NSCLC cells *in vivo* and prolong survival time of tumor-bearing mice. These results demonstrate for the first time that TG101348 enhances the antitumor action of erlotinib in EGFR-mutant and erlotinib-resistant NSCLC cells. TG101348, therefore, may be a potential agent for overcoming erlotinib-resistance in treating NSCLC patients.

In this study, survivin expression was increased in erlotinib-treated H1975 cells. Survivin is a member of inhibitor of apoptosis family. Survivin overexpression has been shown to be associated with tumor progression and drug resistance [41]. Previous studies have shown that PI3K-AKT activation of EGFR signaling upregulates survivin expression, and persistent survivin expression is associated with erlotinib-resistance in EGFR mutation-positive NSCLC cells [42]. Survivin inhibition results in EGFR-TKI-induced apoptosis in EGFR mutation-positive NSCLC cells [17]. In this study, erlotinib did not inhibit survivin expression nor induce apoptosis in H1975 cells, consistent with a previous report [17]. Our results showed that TG101348 significantly inhibited survivin expression and induced apoptosis in H1975 cells. Apoptosis-related proteins bcl-2 family (e.g. Bcl-XL) and Inhibitor of Apoptosis family (e.g. survivin) have also been shown to be linked to erotinib-resistance of NSCLC cells [17]. Bcl-XL and survivin are downstream of both p-STAT3 signaling and EGFR. Thus, the combination of TG101348 and erlotinib markedly induced apoptosis and inhibited survivin and Bcl-XL expression due to inhibition of the activation of both STAT3 and EGFR. Consequently, the combination of TG101348 and erlotinib resulted in the increased induction of apoptosis. Our results are consistent with a previous report that the inhibition of survivin increased erlotinib-induced apoptosis in H1650 cells [17]. The results document that survivin inhibition is one of the mechanisms by which TG101348 enhanced erlotinib anti-tumor effect.

In summary, our results indicate that TG101348 could reverse erlotinib resistance and significantly enhance erlotinib-inhibited cell proliferation and erlotinib-induced apoptosis in erlotinib-resistant NSCLC cells. The inhibition of p-EGFR and p-STAT3 levels as well as survivin expression are main mechanisms by which TG101348 reverse erlotinib resistance in NSCLC cells. Recently, a randomized, placebo-controlled clinical trial showed that healthy volunteers were able to tolerate TG-101348, which could inhibit p-STAT3 in human cells. [43]. Our findings suggest that TG101348 might be a potential adjuvant for NSCLC patients during erlotinib treatment.

## MATERIALS AND METHODS

### Cell culture and reagents

The NSCLC PC-9 (EGFR^19del^), H1650 cells (bearing a deletion in exon 19 of the EGFR gene, i.e., DE746-A750, EGFR^19delE746-A750^) and H1975 cells (EGFR L858R/T790M, EGFR^Exon21L858R+T790M+ Exon20T790M^) (ATCC, Rockville, MD, USA) [31] were maintained in RPMI 1640 medium supplemented with 10% fetal bovine serum (FBS), 100U/ml penicillin and 100 μg/ml streptomycin (Gibco BRL, Life Technologies, NY). TG101348 and erlotinib were purchased from Selleck Chemicals (Houston, TX, USA). For generation of STAT3-knockdown stable cells, H1975 cells were infected with Lentivirus-STAT3-ShRNA particles and Lentivirus-Ctrl-ShRNA particles (Santa Cruz Biotech., Santa Cruz, CA, USA). Stable clones were selected by puromycin and confirmed by Western blot.

### Cell proliferation

Cell proliferation was determined by 3-(4,5-dimethylthiazol-2yl)-2,5-diphenyltetrazolium bromide (MTT) assay. Briefly, exponentially growing NSCLC cells (1 × 10^4^ cells/well) were seeded into 96-well culture plates with 6 replicates. Twenty-four hours later cells were treated with cisplatin, TG101348, erlotinib, or the combination of TG101348 and erlotinib for another 72 hours. MTT (20 μl of 5 mg/ml) were added to each well and incubated for 4 hours at 37°C. Formazan crystals were dissolved by adding 100 μl of dimethyl sulfoxide (DMSO). The color intensity, which is a reflection of the number of live cells, was measured at a wavelength of 570 nm. Growth inhibition is expressed as the percentage of surviving cells in drug-treated cells versus DMSO-treated control cells. The IC50 value is the concentration resulting in 50% cell growth inhibition by a 72 hours exposure to drugs compared with control cells.

### Apoptosis assay

Apoptosis was determined by a terminal deoxynucleotidyl transferase biotin-dUTP nick end labeling (TUNEL) (Roche, Mannheim, Germany) assay according to previous methods [44]. H1975 cells and H1650 cells (1×10^5^ cells/well) were seeded in 6-well plates. Cells were treated with TG101348, erlotinib, or the combination of TG101348 and erlotinib for 48 hours. Cells were fixed by 4% paraformaldehyde for 1 hour and then penetrated with 0.1%Triton X-100 for 15 minutes. TDT enzyme and label solution were added to the fixed cells. After one hour, the cells were observed under a fluorescence microscope (Olympus, Japan). The cells with green nuclei staining were defined as apoptotic cells. The apoptotic cells were assessed in 9 randomly selected fields viewed at 200 × magnification. The apoptotic index was calculated as total number of apoptotic cells in 9 view/total number of nucleated cells ×100%.

### Enzyme-linked immunosorbent assay (ELISA)

NSCLC cells (1 x10^5^) were cultured in 2 ml of RPMI-1640 complete medium in a 6-well plate for 48 hours. Culture media were collected for ELISA. Human IL-6 cytokines in the culture media were quantified using anti-human IL-6 quantitative ELISA kits according to the manufacturer's instructions (R&D, Minneapolis, MN Minneapolis, MN).

### Western blot

Cancer cells treated with erlotinib and TG101348 were lysed with the lysis buffer [50 mm Tris-HCl (pH 7.5), 250 mm NaCl, 0.1% NP40, and 5 mm EGTA containing 50 mm sodium fluoride, 60 mm β-glycerol-phosphate, 0.5 mm sodium vanadate, 0.1 mm phenylmethylsulfonyl fluoride, 10 μg/ml aprotinin, and 10 μg/ml leupeptin]. Protein concentration was measured by using a BCA Protein Assay Kit (Thermo Scientific, Rockford, IL). SDS-polyacrylamide gel electrophoresis (SDS-PAGE) was used to separate proteins. The SDS-polyacrylamide gels were transferred onto PVDF membranes (Millipore, Bedford, MA). The membranes were incubated with specific primary antibodies against EGFR, phospho-EGFR (p-EGFR), STAT3, p-STAT3, Survivin, Bcl-xL, Cleave caspase 3, XIAP, Bcl-2, and β-actin (Cell Signaling Technology, San Diego, CA), and with HRP-conjugated secondary antibodies conjugated to Alexa Fluor 680, or IRdye 800 (Rockland Immunochemicals, Inc. Gilbertsville, PA). The intensities of the protein bands were scanned using the Odyssey Infrared Imaging System (Li-Cor Biosciences, Lincoln, Nebraska).

### Tumor xenograft mouse model

Four-week old female athymic nude mice were bred in a sterilized animal room of the Animal Experimental Centre of Shanghai Institutes for Biological Sciences (Shanghai, China). Animal protocols were approved by Animal Care and Facilities Committee of the Second Affiliated Hospital of Soochow University. H1975 cells (1 x10^6^) were injected subcutaneously (s.c.) into the flanks of athymic nude mice. When tumor size reached approximately 80 mm^3^ −100 mm^3^, mice were randomly assigned to treatment and control groups, with each group of 5 mice. TG101348 was dissolved in propylene glycol, and erlotinib was prepared in drinking water. In the treatment groups, mice received erlotinib (10 mg/kg body weight) [18] and TG101348 (1g/kg body weight) [28] as monotherapy or combination therapy by oral gavage daily for 7 days. The control group received vehicle control. Tumor sizes were measured by a caliper weekly. Five weeks after the treatment, animals were euthanized, and the weight of the tumor was measured. Tumor volume (V) was calculated according to the following equation: V (mm^3^) =1/2 a^2^b(a: relatively shorter diameter, b: relatively longer diameter) [45]. To evaluate the survival time of the xenografted mice, tumor-bearing mice were treated with erlotinib (10 mg/kg body weight) and TG101348 (1g/kg body weight) alone or their combination (10 mice/group) as the same methods above. The end point was until an entire group of mice died. To evaluate the safety of treatment, four important organs of heart, liver, lung and kidney were harvested from the mice treated.

### Immunohistochemistry

Tumor tissues were fixed in 10% formalin. Tumors were embedded in paraffin and stained with hematoxylin and eosin (H&E). The tissue sections were deparaffinized, rehydrated and boiled in 0.01M sodium citrate for antigen retrieval. The endogenous peroxidase activity was quenched. The sections were incubated with anti-cleavage caspase 3 (cell signaling), anti-p-EGFR (1:250, abcam) and anti-p-STAT3 (abcam, Cambridge, MA) overnight at 4°C. Tumor sections were incubated with biotinylated secondary antibodies, streptavidin_biotin complex (Dako, Glostrup, Denmark). Staining was visualized using diaminobenzidine. Representative photos were taken with a Nikon Eclipse E800 microscope equipped with a Nikon DXM1200 digital camera (Nikon instruments, Melville, NY, USA). Cleavage caspase 3, p-EGFR and p-STAT3 staining were recorded as the ratio of positively stained cells to all tumor cells at × 400 magnification.

### Statistical analysis

Data are presented as the means ± SD. The statistical significance of the difference between two groups was evaluated with the Student's t-test or one way variant analysis (ANOVA) by using SPSS15.0 software. P < 0.05 was considered significant. The chi-square test was used to analyze the difference in expression of genes samples. The Kaplan–Meier method was used to analyze the survival time of tumor-bearing mice.

## SUPPLEMENTARY MATERIALS, FIGURES


