# Supplementary material for: JAK2 inhibitor TG101348 overcomes erlotinib-resistance in non-small cell lung carcinoma cells with mutated EGF receptor
Source: Oncotarget. 2015 Mar 29;6(16):14329–43. doi: 10.18632/oncotarget.3685 (PMC4546470; doi:10.18632/oncotarget.3685)
Supplement: Supplementary file 1 [file oncotarget-06-14329-s001.pdf]

JAK2 inhibitor TG101348 overcomes erlotinib-resistance in non-small cell lung carcinoma cells with mutated EGF receptor

Supplementary Material

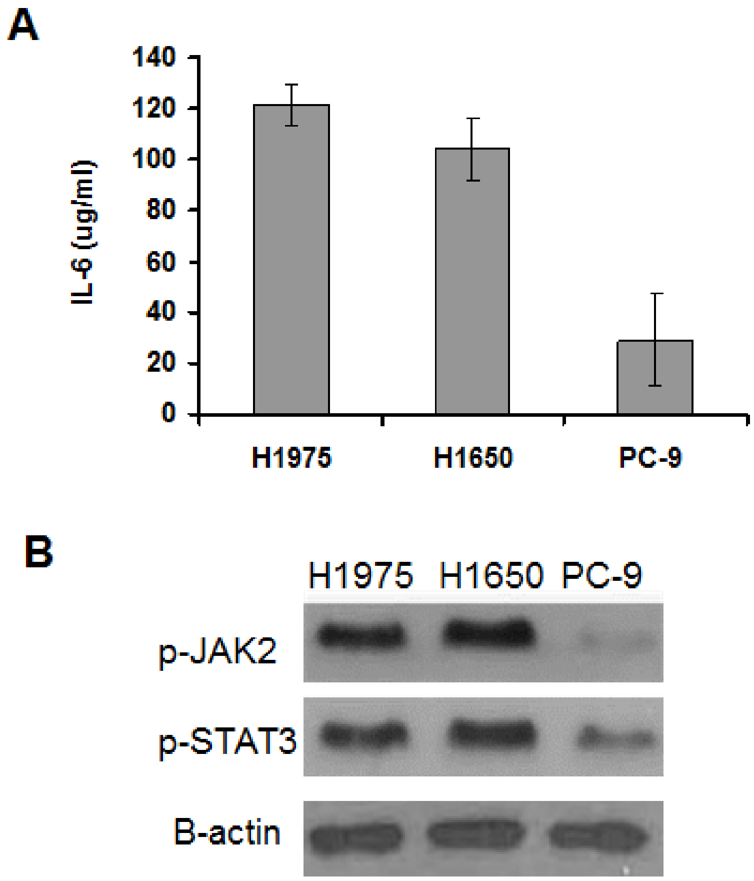

Supplementary Fig. 1. The activation of IL-6/JAK2/STAT3 signaling pathway in erlotinib-resistant NSCLC cells.

(A) NSCLC cells indicated were cultured in RPMI1640 for 48 hours. The culture media were collected and IL-6 levels were determined by IL-6 ELISA kit.

(B) The levels of p-JAK2 and p-STAT3 in indicated cells were determined by Western blot.

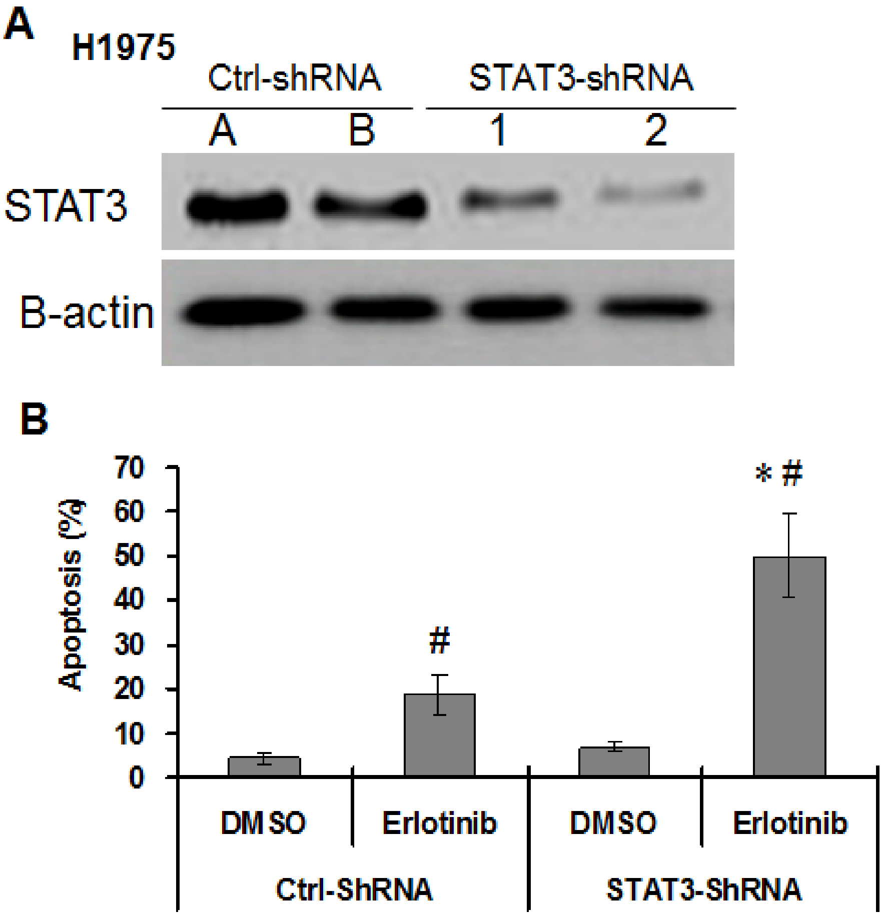

Supplementary Fig. 2. The effect of Knockdown of STAT3 on erlotinib-induced apoptosis in erlotinib-resistant NSCLC.

(A) Generation of H1975 STAT3-Knockdown stable cells. H1975 were infected

Lentivirus-STAT3-shRNA for 48 hours. Cells were selected by puromycin. Western blot confirmed that STAT3 expression were downregulated more 90% in clone 2. The clone 2 was chosen to expand for further study.

(B) Knockdown of STAT3 sensitizes H1975 cells to erlotinib. H1975-ctrl-shRNA (clone A) and H1975 –STAT3-shRNA (clone 2) cells were treated with erlotinib (2.5 μM) for 48 hours. Apoptosis were determined by TUNLE analysis. The data presented are means ± SD of three independent experiments. #p < 0.01, compared to DMSO treatment; *p < 0.01, compared to H1975-Ctrl-shRNA clone.

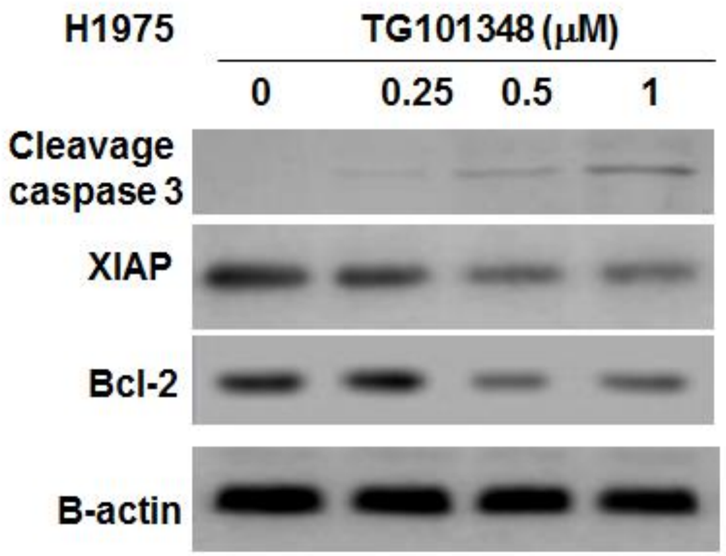

Supplementary Fig. 3. TG101348 inhibits expression of apoptosis-related genes in NSCLC cells. H1975 cells were treated with TG101348 at the indicated doses for 24 hours. Protein expression was determined by Western Blot.

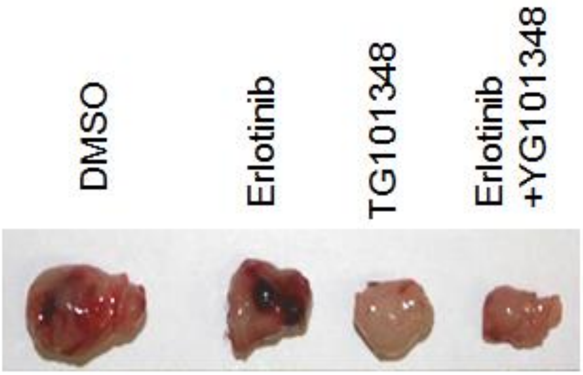

Supplementary Fig. 4 TG101348 potentiates the anti-tumor effect of erlotinib *in vivo*. H1975 cells were injected s.c. into the flanks of athymic nude mice. The tumor-bearing mice received TG101348, erlotinib or their combination treatment for 5 days. Tumor volume was measured weakly for 6 weeks after the treatment. The representative tumor photos were taken in 6th week after the treatment.

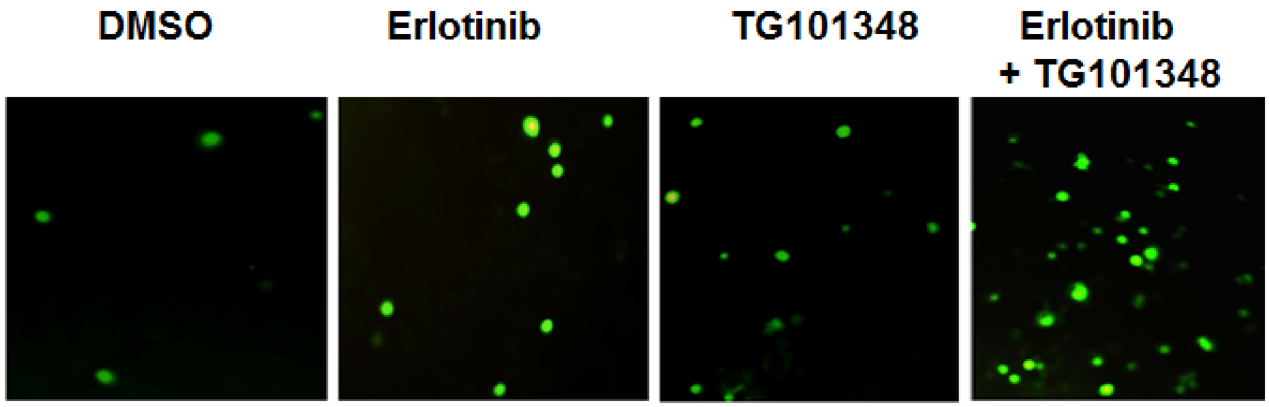

Supplementary Fig. 5. TG101348 enhances erlotinib-induced apoptosis in NSCLC cells. H1975 cells were treated with TG101348 (1 μM), erlotinib (2.5 μM), or a combination of TG101348 (1 μM) and erlotinib (2.5 μM) for 48 hours. Apoptosis was determined by TUNEL. The representative photos of TUNEL were shown.
